# Targeting MCM2 function as a novel strategy for the treatment of highly malignant breast tumors

**DOI:** 10.18632/oncotarget.5408

**Published:** 2015-09-30

**Authors:** Shinya Abe, Kouhei Yamamoto, Morito Kurata, Shiho Abe-Suzuki, Rie Horii, Futoshi Akiyama, Masanobu Kitagawa

**Affiliations:** ^1^ Department of Comprehensive Pathology, Graduate School of Medical and Dental Sciences, Tokyo Medical and Dental University, Tokyo, Japan; ^2^ Department of Pathology, Cancer Institute Hospital, Japanese Foundation for Cancer Research, Tokyo, Japan; ^3^ Department of Pathology, Cancer Institute, Japanese Foundation for Cancer Research, Tokyo, Japan

**Keywords:** apoptosis, breast cancer, cancer stem cell, DNA-damage, MCM2

## Abstract

Highly malignant tumors express high levels of the minichromosome maintenance 2 (MCM2) protein, which is associated with advanced tumor grade, advanced stage, and poor prognosis. In a previous study, we showed that Friend leukemia virus (FLV) envelope protein gp70 bound MCM2, impaired its nuclear translocation, and enhanced DNA-damage-induced apoptosis in FLV-infected hematopoietic cells when the cells expressed high levels of MCM2. Here, we show that MCM2 is highly expressed in clinical samples of invasive carcinoma of the breast, especially triple-negative breast cancer (TNBC), and in cancer stem cell (CSC) marker-positive breast cancer cells. To generate a cancer therapy model using gp70, we introduced the gp70 protein into the cytoplasm of murine breast cancer cells that express high levels of MCM2 by conjugating the protein transduction domain (PTD) of Hph-1 to gp70 (Hph- 1-gp70). Hph-1-gp70 was successfully transduced into the cytoplasm of breast cancer cells. The transduced protein enhanced the DNA damage-induced apoptosis of cancer cells *in vitro* and *in vivo*. Therefore, an MCM2-targeted strategy using Hph-1-gp70 treatment to induce DNA damage might be a successful therapy for highly malignant breast cancers such as TNBC and for the eradication of CSC-like cells from breast cancer tissue.

## INTRODUCTION

Invasive carcinoma, a common breast malignancy, is a major cause of cancer-related deaths in women worldwide [1]. Breast cancer is a heterogeneous disease. Molecularly distinct subtypes have different characters of biological behaviors and prognoses [2]. Immunohistochemical evaluation of estrogen receptor (ER), progesterone receptor (PgR), Ki-67, and human epidermal growth factor receptor 2 (HER2) is commonly used to identify the four molecular subtypes: HER2-type, luminal-type, luminal/HER2-type, and triple negative (TN)-type. Importantly, the subtypes have different clinical outcomes [3–6]. In particular, TN breast cancer (TNBC) is associated with an increased risk of early relapse, usually within 5 years from the initial diagnosis [7]. Unlike tumors that overexpress hormone receptors and HER2, TNBC cannot be treated with hormone therapy or trastuzumab, an anti-HER2 monoclonal antibody, because TNBC tumor cells lack these specific targets [8, 9]. Consequently, patients with TNBC have worse outcomes after chemotherapy than patients with other breast cancer subtypes [10]. Thus, novel therapies that target highly malignant TNBC are needed to improve breast cancer prognosis.

Cancer stem cells (CSCs) are thought to contribute to tumor initiation and maintenance, chemotherapy resistance, tumor relapse, and metastasis [11–14]. Therefore, for the successful treatment of many types of cancer, strategies that target CSCs are also needed. Multiple markers such as CD13, CD44, delta-like homologue 1, CD133, and aldehyde dehydrogenase 1 (ALDH-1) have been used to isolate and identify CSCs in various tumors [15–18]. In particular, CD44, CD133, and ALDH-1 have been used as CSC markers for the enrichment of CSCs in breast cancer [19–21].

Minichromosome maintenance (MCM) 2 is one of six related proteins that comprise the MCM complex (MCM2–7), which has an essential role in DNA replication [22]. Previous studies with human samples have established MCM2 as a proliferation marker. A high level of MCM2 expression in malignant tumors is associated with several clinicopathological parameters such as advanced tumor grade, advanced stage, and poor prognosis [23–26]. Previously, we showed that Friend leukemia virus (FLV) infection markedly enhanced the irradiation (IR)-induced apoptosis of hematopoietic cells in C3H mice expressing high levels of MCM2 via the activation of ATM, DNA-PK, and P53 [27]. Apoptosis was enhanced almost exclusively in the C3H strain [28]. We also demonstrated that the FLV-derived envelope protein gp70 enhanced cellular apoptotic signaling specifically in cells that overexpressed MCM2 [29]. Gp70 bound directly to the nuclear localization signal of MCM2 and inhibited its nuclear translocation. The cytoplasmic MCM2-gp70-complex bound to protein phosphatase 2A (PP2A), interfered with the PP2A-DNA-PK interaction, and enhanced DNA damage-induced apoptosis via the activation of p53 by DNA-PK [30]. These results suggest that regulation of the molecular dynamics of MCM2 by using gp70 offers a novel therapeutic approach by which malignant tumors that express high levels of MCM2 can be specifically eliminated. To develop an MCM2-targeted therapy, a method for efficiently introducing gp70 protein into cancer cells is required.

We focused on a protein delivery system that uses the protein transduction domain (PTD). PTD is a peptide sequence that can penetrate the cell membrane [31–33]. PTD has been widely used as a carrier for the delivery of various proteins into living cells. Protein transduction therapy using PTD for the intracellular delivery of a protein is an alternative approach to viral gene therapy [34]. Recently, a novel PTD was identified in the human transcription factor Hph-1. The Hph-1 PTD has been used to deliver immunosuppressive proteins *in vitro* and *in vivo* for the treatment of autoimmune diseases [35, 36].

In the present study, we analyzed the expression of MCM2 in HER2, luminal, luminal/HER2, and TN subtypes of human breast cancer. Furthermore, we introduced gp70 into murine breast cancer cells using PTD and investigated whether gp70 had apoptosis-enhancing effects in solid tumors such as breast cancers.

## RESULTS

### MCM2 is highly expressed in triple negative breast cancer

**Figure 1:**
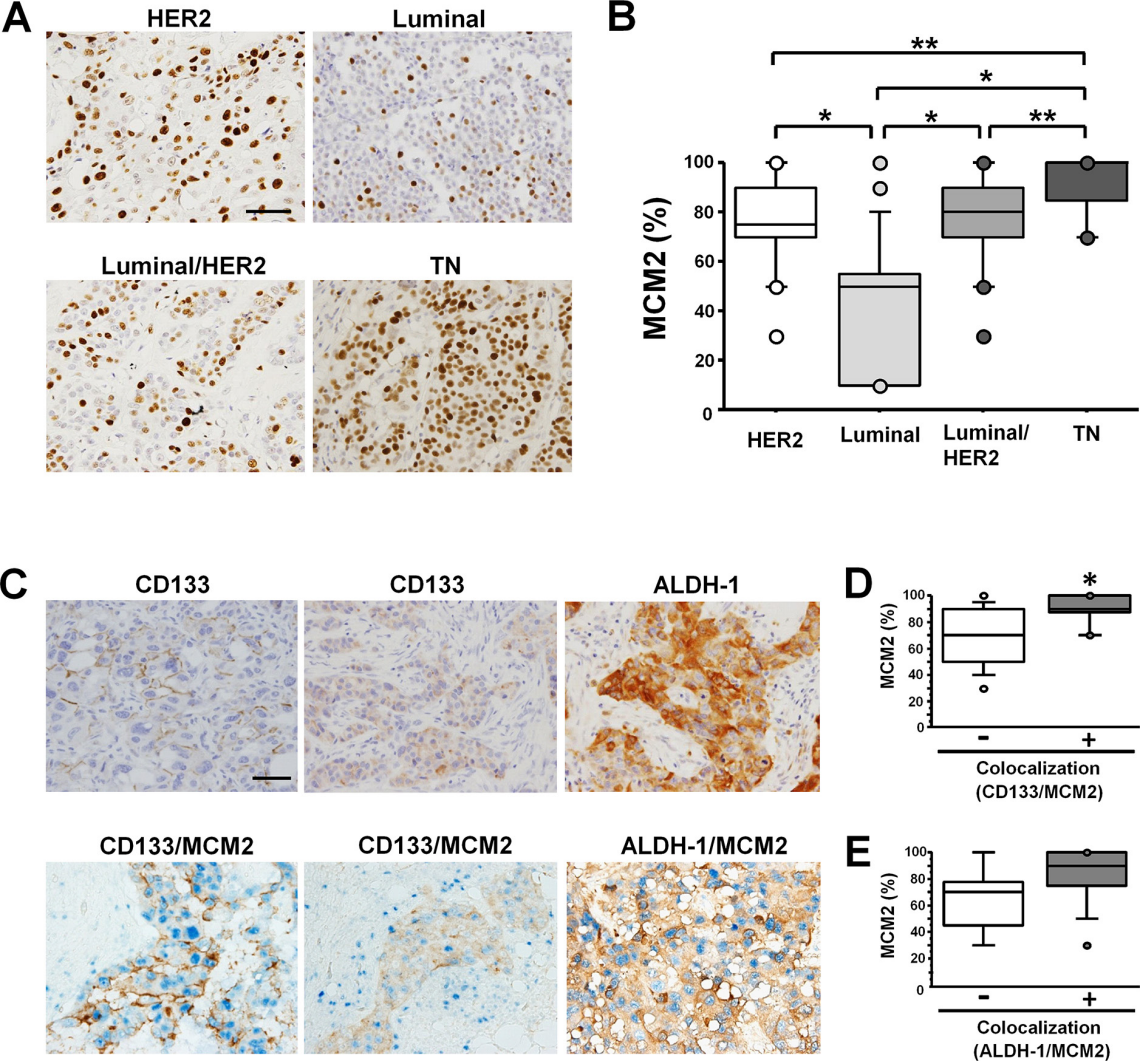
In samples of invasive carcinoma of no special type, MCM2 expression is highest and MCM2 frequently colocalizes with CSC markers in TNBC specimens **A.** MCM2 immunohistochemical staining of invasive carcinomas of no special type showing representative features of the HER2 group (top-left), luminal group (top-right), luminal/HER2 group (bottom-left), and TN group (bottom-right). Scale bar indicates 100 μm. Note the frequent nuclear signals in the TNBC case. **B.** Labeling index of MCM2 in each breast cancer subtype. The index of the TN type (*n* = 20) of breast cancer was significantly higher than the indices of the HER2 (*n* = 30), luminal (*n* = 25), and luminal/HER2 (*n* = 29) types. *P < 0.0001, **P < 0.01 by Mann-Whitney *U* test. **C.** Immunostaining for CD133 in cases with invasive carcinoma of no special type. CD133 antigen localized to the cell membrane (top-left) or the cytoplasm (top-middle). Immunostaining for ALDH-1 in breast cancer (top-right). Double immunostaining for CD133 (brown) and MCM2 (blue) (bottom-left, middle). Double immunostaining for ALDH-1 (brown) and MCM2 (blue) (bottom-right). Scale bar indicates 100 μm. **D.** The labeling index of MCM2 in the CD133/MCM2 colocalized group (*n* = 17) and non-colocalized group (*n* = 10). *P < 0.01 by Mann-Whitney *U* test. **E.** The labeling index of MCM2 in the ALDH-1/MCM2 colocalized group (*n* = 15) and non-colocalized group (*n* = 5).

To quantify MCM2 protein expression in each breast cancer subtype, immunohistochemical staining was performed using specimens from human cases with invasive carcinoma of no special type (Figure 1A). The labeling index of MCM2 in the TN group was significantly higher than the indices in all other subtype groups (Figure 1B). The MCM2 labeling index of the HER2 and luminal/HER2 groups was higher than that of the luminal group (Figure 1B).

### Cancer stem cell markers are frequently expressed in TNBC and colocalize with MCM2

Two patterns of CD133 expression were identified: staining of the cell membrane (Figure 1C, top-left) and staining of the cytoplasm (Figure 1C, top-middle). In contrast, ALDH-1 was exclusively expressed in the cytoplasm (Figure 1C, top-right). The frequency of CD133-positive cases was highest in the TN group (40.0%, 8 of 20), followed by the HER2 group (30.0%, 9 of 30), luminal/HER2 group (27.6%, 8 of 29), and luminal group (8.0%, 2 of 25) (Table 1). The membrane staining pattern of CD133 was most frequently observed in the TN group (37.5%), followed by the HER2 group (22.2%), luminal/HER2 group (12.5%), and luminal group (0%). However, the cytoplasmic staining pattern of CD133 was most frequently observed in the luminal group (100%), followed by the luminal/HER2 group (87.5%), HER2 group (77.8%), and TN group (62.5%) (Table 1). The percentage of ALDH-1-positive cases was highest in the TN group (25.0%, 5 of 20), followed by the HER2 group (23.3%, 7 of 30), luminal/HER2 group (17.2%, 5 of 29), and luminal group (12.0%, 3 of 25) (Table 1).

**Table 1:** Cancer stem cell marker was frequently expressed in TNBC and was co-localized with MCM2

	CD133 positive	Staining pattern		CD133 & MCM2		ALDH-1 positive	ALDH-1 & MCM2	
		cell membrane	cytoplasm	Co-localized	Non co-localized		Co-localized	Non co-localized
**HER2 (*n* = 30)**	9 (30.0%)	2 (22.2%)	7 (77.8%)	6 (66.7%)	3 (33.3%)	7 (23.3%)	7 (100%)	0 (0%)
**Luminal (*n* = 25)**	2 (8.0%)	0 (0%)	2 (100%)	0 (0%)	2 (100%)	3 (12.0%)	0 (0%)	3 (100%)
**Luminal/HER2 (*n* = 29)**	8 (27.6%)	1 (12.5%)	7 (87.5%)	4 (50.0%)	4 (50.0%)	5 (17.2%)	5 (100%)	0 (0%)
**TN (*n* = 20)**	8 (40.0%)	3 (37.5%)	5 (62.5%)	7 (87.5%)	1 (12.5%)	5 (25.0%)	3 (60.0%)	2 (40.0%)

Next, we performed double immunostaining for CD133 or ALDH-1 and MCM2 in cases that were positive for CSC markers (Figure 1C, bottom). Colocalization of MCM2 and CD133 was most frequently observed in the TN group (87.5%, 7 of 8), followed by the HER2 group (66.7%, 6 of 9), luminal/HER2 group (50.0%, 4 of 8), and luminal group (0%, 0 of 2) (Table 1). The labeling index of MCM2 was significantly higher in the CD133/MCM2 colocalized group than in the non-colocalized group (Figure 1D). Colocalization of MCM2 and ALDH-1 was most frequently observed in the HER2 (100%, 7 of 7) and luminal/HER2 (100%, 5 of 5) groups, followed by the TN group (60.0%, 3 of 5) and luminal group (0%, 0 of 3) (Table 1). The labeling index of MCM2 was higher in the ALDH-1/MCM2 colocalized group than in the non-colocalized group, although the difference was not statistically significant (Figure 1E). These results suggest that the CSC markers are highly expressed in TNBC cases and that the markers frequently colocalize with MCM2.

### Direct introduction of Hph-1-conjugated gp70 into breast cancer cells *in vitro*

**Figure 2:**
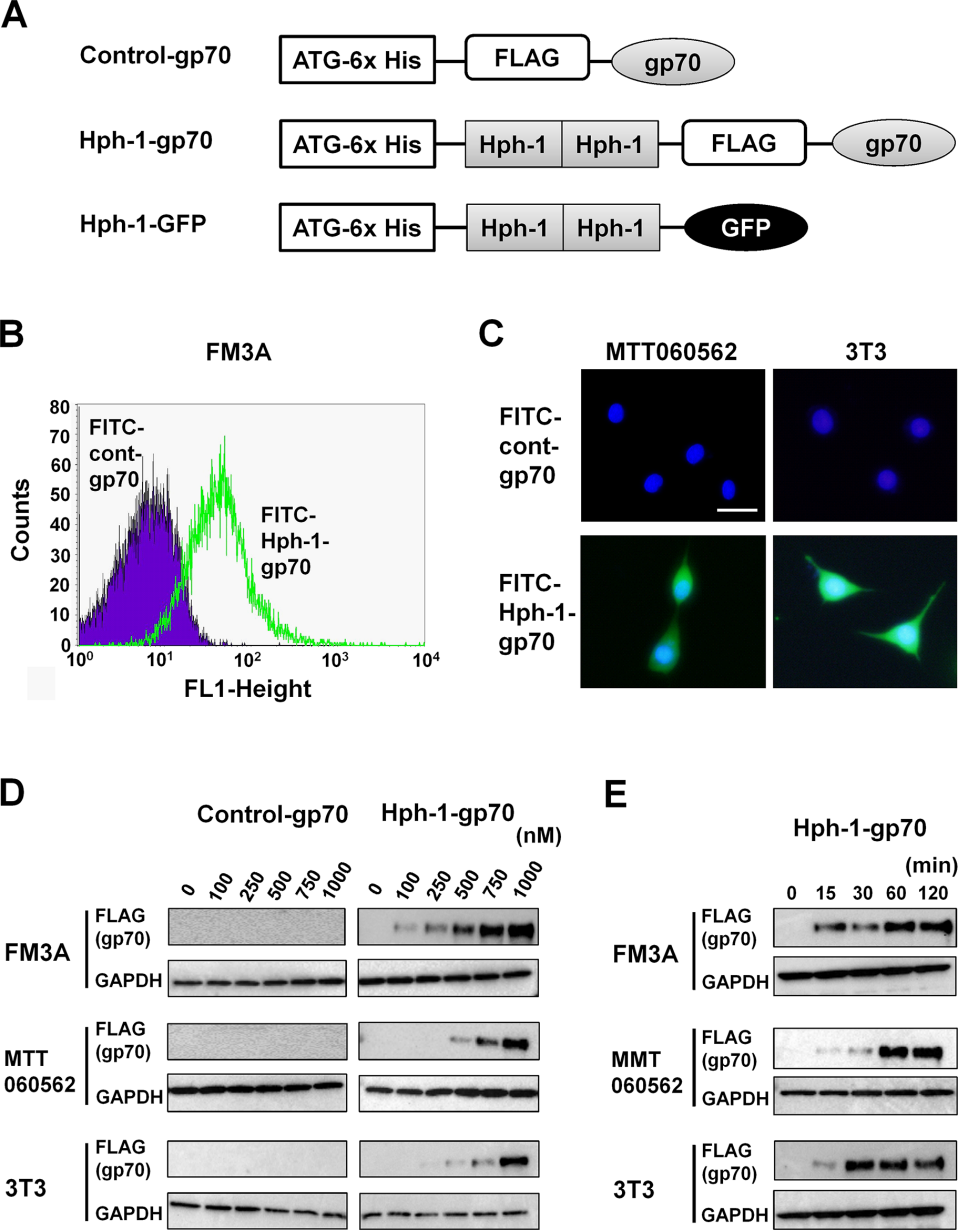
Protein transduction domain Hph-1 conjugated gp70 protein was directly transduced into the cell **A.** Schematic illustration indicating the structure of control-gp70, Hph-1-gp70, and Hph-1-GFP. **B.** FITC-conjugated control-gp70 or Hph-1-gp70 were introduced into 1 × 10^6^ FM3A at 1 μM for 2 h. FITC-positive FM3A cells were analyzed by FACS. **C.** FITC-conjugated control-gp70 or Hph-1-gp70 were introduced into 1 × 10^4^ MTT060562 (left) and 3T3 (right) at 1 μM for 2 h. Hoechst-stained nuclei are shown in blue. FITC-positive cells were acquired using a BZ-9000 microscope (KEYENCE) with a 100 × objective. Scale bar indicates 100 μm. **D.** Control-gp70 or Hph-1-gp70 were introduced into 1 × 10^6^ FM3A, MTT060562, and 3T3 cells at concentrations of 0, 100, 250, 500, 750, and 1000 nM for 2 h. The expression of exogenous gp70 was verified with western blot analysis. **E.** Hph-1-gp70 was introduced into 1 × 10^6^ FM3A, MTT060562, and 3T3 cells at 1 μM for 0, 15, 30, 60, and 120 min, and the expression of exogenous gp70 was verified with western blot analysis.

To identify the breast cancer subtype of the cells, the expression of *ER*, *PgR*, and *Her2* in FM3A and MTT060562 cells was examined using quantitative RT-PCR. Although their expression levels of ER, PgR, and Her2 were much lower than those of the control uterine tissue, FM3A and MTT060562 cells exhibited higher expression levels of ER and Her2 than 3T3 cells. FM3A cells had higher expression of PgR than 3T3 cells (Figure S1). To investigate the Hph-1-mediated transduction of gp70 into murine breast cancer FM3A and MTT060562 cells, we generated DNA constructs and expressed and purified control-gp70, Hph-1-gp70, and Hph-1-GFP (Figure 2A). To introduce the recombinant proteins into the cells, the FITC-conjugated recombinant proteins were incubated with FM3A, MTT060562 and 3T3 cells at a concentration of 1 μM for 2 h. Hph- 1-gp70 was transduced into the living FM3A (Figure 2B), MTT060562 and 3T3 cells (Figure 2C). To determine whether lower concentrations of the recombinant proteins could be efficiently transduced into the cells, 1 × 10^6^ FM3A and MTT060562 murine breast cancer cells and 3T3 murine fibroblast cells were incubated with the proteins at concentrations of 0, 100, 250, 500, 750, and 1000 nM for 2 h. The transduction of gp70 was then assessed using western blot analysis (Figure 2D). Hph-1-gp70 was transduced into the cytoplasm of the cell lines in a concentration-dependent manner. Transduced gp70 was detected in FM3A cells incubated with ≥ 100 nM of Hph-1-gp70 and in MTT060562 and 3T3 cells incubated with ≥ 500 nM of Hph-1-gp70 (Figure 2D). To measure the time course of Hph-1-gp70 transduction, 1 μM of Hph-1-gp70 was incubated with 1 × 10^6^ FM3A, MTT060562, and 3T3 cells for 0, 15, 30, 60, and 120 min, and gp70 transduction was assessed with western blot analysis (Figure 2E). Hph-1-gp70 was first detected at 15 min, and the maximum concentration was reached in less than 60 min in FM3A, MTT060562, and 3T3 cells (Figure 2E). These results suggest that Hph-1-gp70 is rapidly introduced into the cytoplasm of the various cells tested.

### Hph-1-gp70 binds MCM2 directly

**Figure 3:**
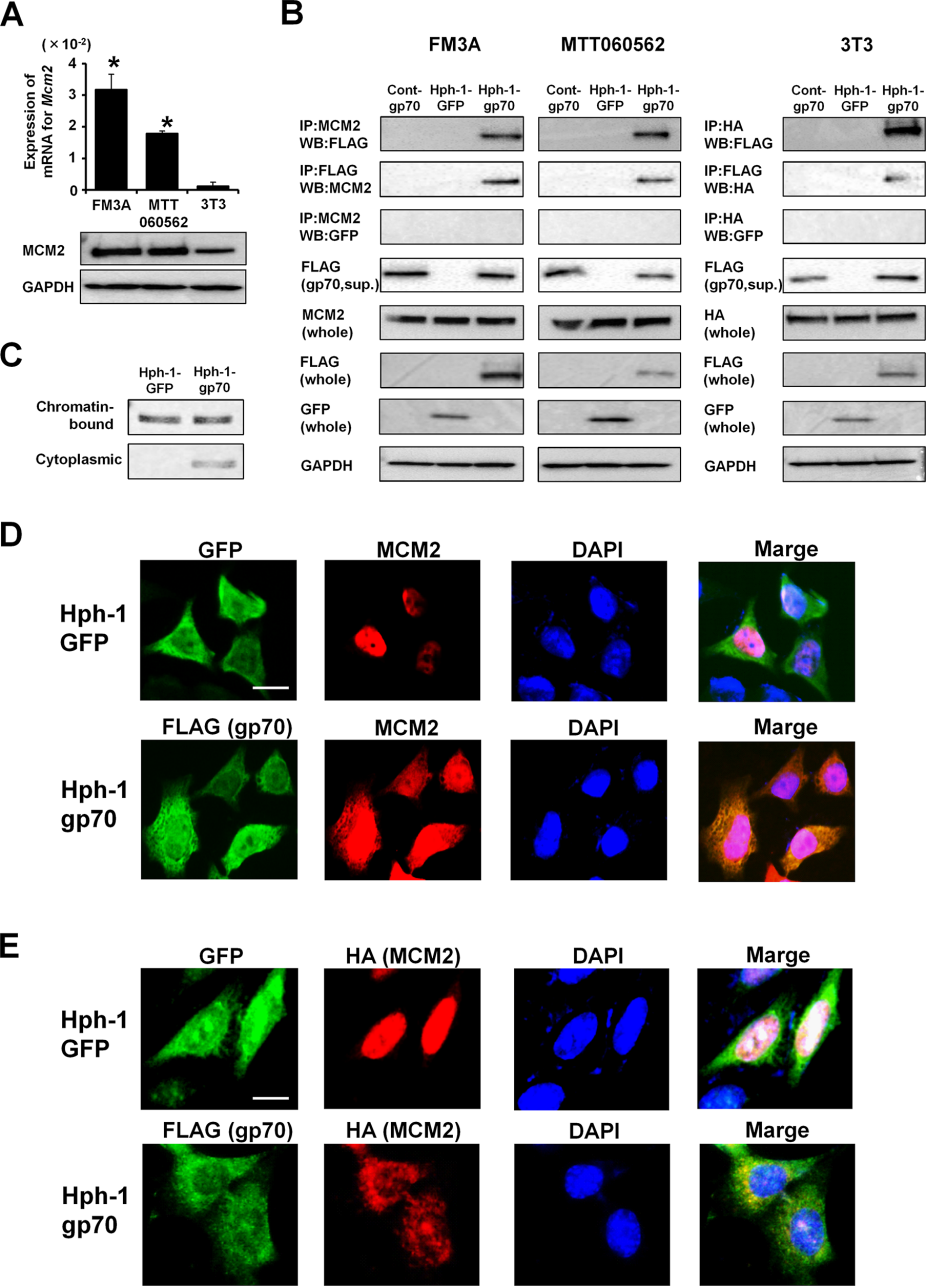
Hph-1-gp70 binds MCM2 directly **A.** Quantitative RT-PCR analysis of *Mcm2* mRNA expression (top) and western blotting analysis of MCM2 (bottom) in FM3A, MTT060562, and 3T3 cells. *P < 0.01 vs. 3T3 cells by two-tailed Student's *t*-test. **B.** FM3A (left), MTT060562 (middle), and *HA-Mcm2*-expressing 3T3 cells (right) were treated with 1 μM control-gp70, Hph-1-GFP, or Hph- 1-gp70 for 2 h. Cell lysates were subjected to pull-down assays to assess the binding of MCM2 to gp70. **C.** FM3A cells were treated with 1 μM Hph-1-GFP or Hph-1-gp70 for 2 h. Cell lysates from these cells were separated into chromatin-bound and cytoplasmic fractions. MCM2 protein was analyzed with western blotting. **D.** MTT060562 cells were treated with 1 μM Hph-1-GFP or Hph-1-gp70 for 2 h. FLAG-positive cells containing the gp70-derived proteins are shown in green (FITC). Endogenous MCM2-positive cells are shown in red (TRITC). DAPI-stained nuclei are shown in blue. Images were acquired using a BZ-9000 microscope (KEYENCE) with a 400 × objective. Scale bar indicates 25 μm. **E.** *HA-Mcm2*-expressing 3T3 cells were treated with 1 μM Hph-1-GFP or Hph-1-gp70 for 2 h. FLAG-positive cells containing the gp70-derived proteins are shown in green (FITC). HA-positive cells containing the MCM2-derived proteins are shown in red (TRITC). DAPI-stained nuclei are shown in blue. Images were acquired using a BZ-9000 microscope (KEYENCE) with a 400 × objective. Scale bar indicates 25 μm.

The basal levels of endogenous MCM2 mRNA and protein were significantly higher in FM3A and MTT060562 cells than in 3T3 cells (Figure 3A). We next examined the intracellular interaction between Hph-1-gp70 and MCM2. Control-gp70, Hph-1-gp70, or Hph-1-GFP (1 μM) was incubated with 1 × 10^6^ FM3A, MTT060562, and 3T3 cells for 2 h. Immunoprecipitation experiments revealed that Hph-1-gp70 bound to endogenous MCM2 in FM3A and MTT060562 cells (Figure 3B), but not in 3T3 cells (data not shown). However, Hph-1-gp70 bound to MCM2 in 3T3 cells when the cells were transfected with an expression plasmid encoding HA-conjugated MCM2 (Figure 3B).

In Hph-1-GFP-treated FM3A cells, MCM2 was not detected in the cytoplasmic fractions. By contrast, MCM2 was detected in the cytoplasmic fraction of FM3A cells treated with Hph-1-gp70 (Figure 3C). Furthermore, MCM2 localized to the nucleus of Hph-1-GFP-treated MTT060562 (Figure 3D, top) and *HA-Mcm2*-expressing 3T3 cells (Figure 3E, top). However, MCM2 remained in the cytoplasm of Hph-1-gp70-treated cells, in which Hph-1-gp70 colocalized with endogenous MCM2 in MTT060562 cells (Figure 3D, bottom) and HA-MCM2 in 3T3 cells (Figure 3E, bottom).

### Hph-1-gp70 enhances DNA damage-induced apoptosis

**Figure 4:**
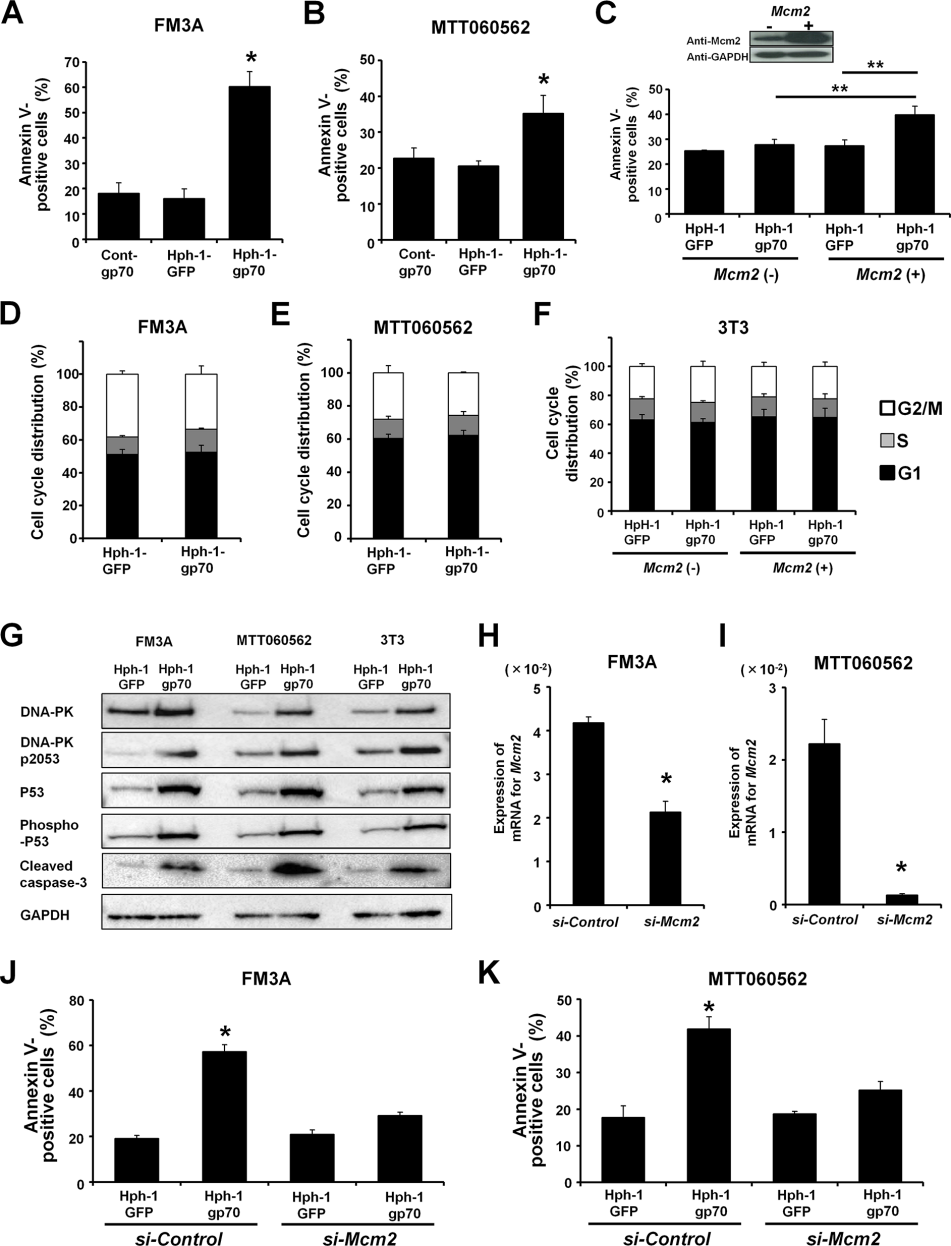
Hph-1-gp70 enhances doxorubicin-induced apoptosis **A.** FM3A and **B.** MTT060562 cells were treated with 1 μM cont-gp70, Hph-1-GFP, or Hph-1-gp70 and 500 nM doxorubicin, and the apoptotic cell ratios were determined 24 h later with annexin V-staining. **C.** 3T3 cells transfected with *HA*-empty vector or *HA-Mcm2* were treated with 1 μM Hph-1-GFP or Hph-1-gp70 and 500 nM doxorubicin, and the apoptotic cell ratios were determined 24 h later with annexin V-staining. **D.** FM3A and **E.** MTT060562 cells were treated with 1 μM Hph-1-GFP or Hph-1-gp70 for 24 h. The effects of treatment on the cell cycle profiles were analyzed with FACS. **F.** 3T3 cells transfected with *HA*-empty vector or *HA-Mcm2* were treated with 1 μM Hph-1-GFP or Hph-1-gp70 for 24 h. The effects on the cell cycle profiles were analyzed with FACS. **G.** Representative western blots for DNA-PK, phospho-DNA-PK (pS2053), P53, phospho-P53, and cleaved caspase-3 in FM3A, MTT060562, and *HA-Mcm2*-transfected 3T3 cells. The cells were treated with 1 μM Hph-1-GFP or Hph-1-gp70 and 500 nM doxorubicin for 24 h. **H.** *Mcm2* knockdown in FM3A and **I.** MTT060562 cells by using siRNA. Quantitative RT-PCR was performed to confirm the *si-Mcm2*-induced reduction in *Mcm2* mRNA expression. **J.** FM3A and **K.** MTT060562 cells transduced with *si-control* or *si-Mcm2* were treated with 1 μM Hph-1-GFP or Hph-1-gp70 and 500 nM of doxorubicin, and the apoptotic cell ratios were determined 24 h later with annexin V-staining. *P < 0.01, **P < 0.05 by two-tailed Student's *t*-test.

After treatment with Hph-1-gp70, the apoptotic cell ratio increased in FM3A and MTT060562 cells, which express high levels of endogenous *Mcm2* compared to control-gp70. In contrast, the apoptotic ratio did not increase when the cells were treated with Hph-1-GFP (Figure 4A and 4B). However, treatment with Hph-1-gp70 did not significantly change the apoptotic cell ratio of 3T3 cells, which express low levels of endogenous *Mcm2* (Figure 4C). As expected, the apoptotic cell ratio of 3T3 cells overexpressing HA-MCM2 was higher after treatment with Hph-1-gp70 than after treatment with Hph-1-GFP (Figure 4C). Moreover, we confirmed that treatment with Hph- 1-GFP or Hph-1-gp70 did not cause any significant changes in the cell cycle profiles of FM3A (Figure 4D), MTT060562 (Figure 4E), and 3T3 cells (Figure 4F). These results indicate that a certain expression level of MCM2 is required for the enhancement of apoptosis by gp70.

As shown in Figure 4G, FM3A, MTT060562, and *HA-Mcm2*-expressing 3T3 cells treated with Hph-1-gp70 and doxorubicin expressed higher levels of DNA-PK, phospho-DNA-PK, P53, phospho-P53, and cleaved caspase-3 compared to cells treated with Hph- 1-GFP and doxorubicin.

Treatment with siRNA (*si-Mcm2*) significantly reduced the expression of *Mcm2* mRNA in FM3A and MTT060562 cells (Figure 4H and 4I). In contrast to the increased apoptotic cell ratios observed in FM3A and MTT060562 cells treated with *si-Control*, the apoptotic cell ratios in FM3A and MTT060562 cells treated with *si-Mcm2* did not change markedly after Hph-1-gp70 and doxorubicin treatment (Figure 4J and 4K). Taken together, these results suggest that Hph-1-gp70 bound directly to MCM2, inhibited the translocation of MCM2 to the nucleus by forming a cytoplasmic MCM2-gp70 complex, and strongly enhanced the frequency of doxorubicin-induced apoptosis.

### Hph-1-gp70 and MCM2 complex binds to PP2A and causes hyperphosphorylation of DNA-PK

**Figure 5:**
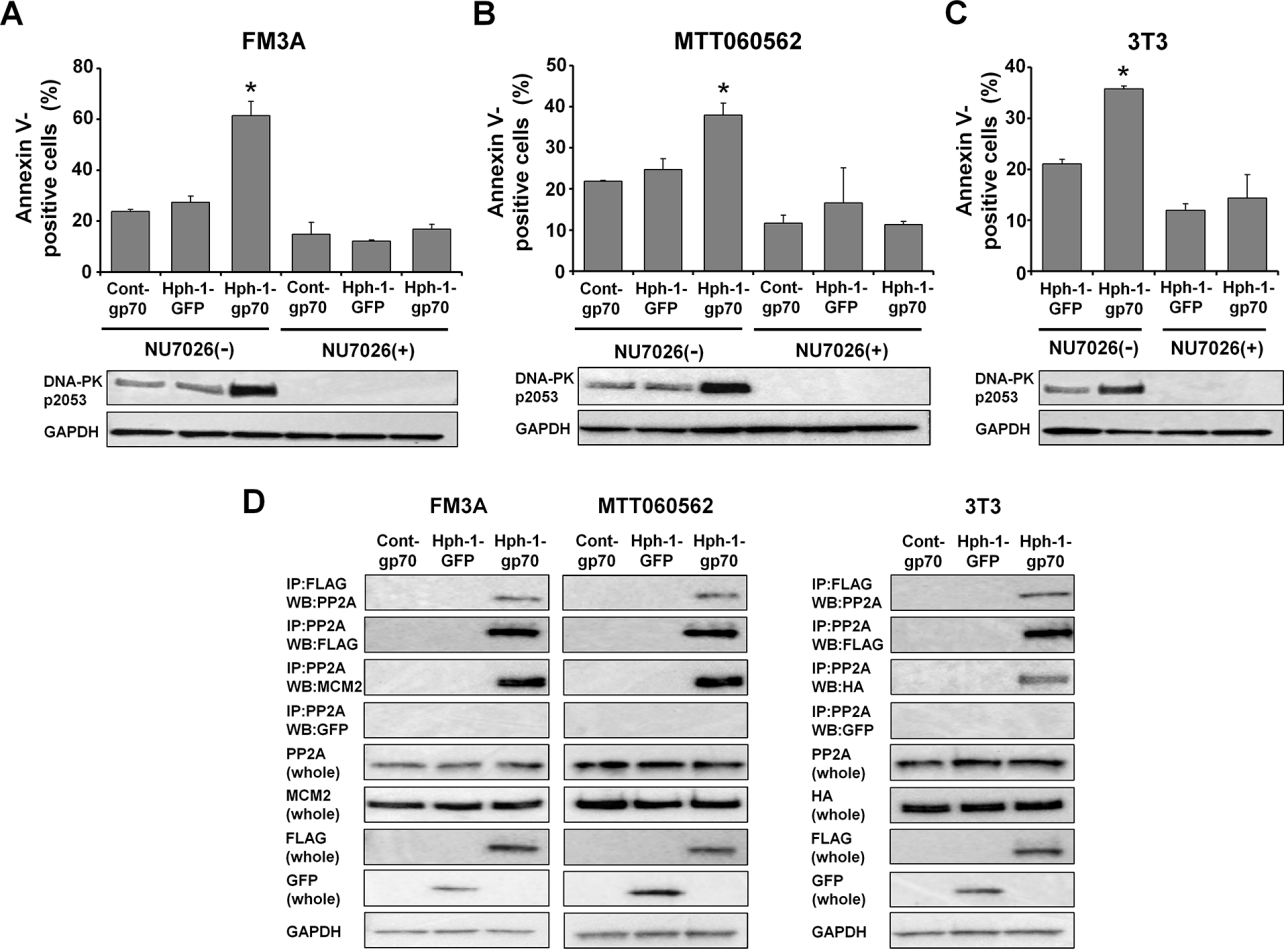
The Hph-1-gp70-MCM2 complex binds to PP2A and causes hyperphosphorylation of DNA-PK **A.** FM3A cells and **B.** MTT060562 cells were pre-incubated with or without 10 μM NU7026, a DNA-PK-inhibitor, for 2 h and treated with 1 μM cont-gp70, Hph-1-GFP, or Hph-1-gp70 and 500 nM doxorubicin for 24 h. The apoptotic cell ratios were determined 24 h later with annexin V-staining (top). *P < 0.01 by two-tailed Student's *t*-test. DNA-PK-pS2053 levels were analyzed by western blotting (bottom). **C.** *HA-Mcm2*-expressing 3T3 cells were pre-incubated with or without 10 μM NU7026 for 2 h and treated with 1 μM Hph-1-GFP or Hph-1-gp70 and 500 nM doxorubicin for 24 h. The apoptotic cell ratios were determined 24 h later with annexin V-staining (top). *P < 0.01 by two-tailed Student's *t*-test. DNA-PK-pS2053 levels were analyzed by western blotting (bottom). **D.** FM3A (left), MTT060562 (middle), and *HA-Mcm2*-transfected 3T3 cells (right) were treated with 1 μM cont-gp70, Hph-1-GFP, or Hph-1-gp70 for 2 h. Cell lysates were subjected to pull-down assays to assess the binding of PP2A to gp70 and PP2A to MCM2.

To examine whether DNA-PK was exclusively required for the enhancement of apoptosis by gp70, we inhibited DNA-PK activity using the drug NU7026 in FM3A, MTT060562 and *HA-Mcm2*-expressing 3T3 cells treated with control-gp70, Hph-GFP, or Hph-1-gp70 and doxorubicin. Inhibition of DNA-PK activity by NU7026 substantially reduced the levels of phospho-DNA-PK (pS2053) and completely abolished apoptosis enhancement in Hph-1-gp70-treated FM3A (Figure 5A), MTT060562 (Figure 5B), and *HA-Mcm2*-expressing 3T3 cells (Figure 5C). These results indicate that DNA-PK activation is necessary for the enhancement of apoptosis by Hph- 1-gp70. In a previous study, we showed that the gp70-MCM2 complex bound to and inhibited protein phosphatase 2A (PP2A). Therefore, we next determined whether the Hph-1-gp70 and MCM2 complex bound to PP2A. FM3A, MTT060562, and *HA-Mcm2*-expressing 3T3 cells were treated with control-gp70, Hph- 1-GFP, or Hph-1-gp70. In Hph-1-gp70 treated cells, PP2A co-precipitated with Hph-1-gp70 and MCM2 (Figure 5D). These results suggest that the Hph-1-gp70 and MCM2 complex bound to PP2A.

### Treatment with Hph-1-gp70 and doxorubicin enhances cancer cell apoptosis and improves the survival of tumor-bearing mice *in vivo*

**Figure 6:**
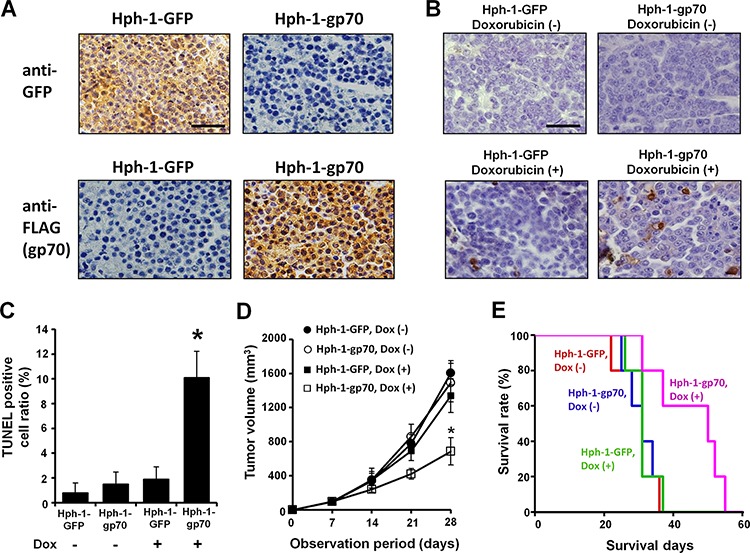
*In vivo* anti-tumor effects of Hph-1-gp70 and DNA damage in FM3A cells in SCID mice **A.** Seven days after the transplantation of FM3A cells, mice were treated with Hph-1-gp70 or Hph-1-GFP and 1.5 mg/kg of doxorubicin or PBS. Expression of GFP (upper) and gp70 (lower) in FM3A tumor samples from Hph-1-gp70- or Hph-1-GFP-injected mice examined with immunohistochemistry. Images were captured with a microscope at 400 × magnification. Scale bar indicates 100 μm. **B.** Microscopic features of TUNEL-positive cells in an FM3A tumor. Scale bar indicates 100 μm. **C.** TUNEL-positive cell ratio in each group of mice. *P < 0.01 by two-tailed Student's *t*-test. **D.** To evaluate the therapeutic effect of Hph-1-gp70 and low-dose doxorubicin, FM3A cells were transplanted into SCID mice. Seven days after the transplantation, mice were treated with 5 mg Hph-1-gp70 or Hph-1-GFP and 1.5 mg/kg of doxorubicin or PBS twice a week. The tumor size in each mouse was assessed once a week. *P < 0.05 by two-tailed Student's *t*-test. **E.** Kaplan-Meier survival curves for FM3A-transplanted SCID mice treated with Hph-1-gp70 or Hph-1-GFP and doxorubicin. The survival time of the Hph-1-gp70, doxorubicin (+) group was longer than that of the other groups (*P* < 0.01 by log-rank test).

To determine whether breast cancer FM3A cells exhibit enhanced apoptosis in response to Hph-1-gp70 and DNA damage *in vivo*, FM3A cells were subcu- taneously transplanted into SCID mice. The mice were intraperitoneally injected with Hph-1-GFP or Hph-1-gp70 and treated with doxorubicin after the FM3A tumor mass was established. As expected, the FM3A tumor samples from Hph-1-gp70-injected mice exhibited strong expression of gp70 (Figure 6A, bottom-right), whereas no gp70 expression was detected in Hph-1-GFP-injected mice (Figure 6A, top-right), which exhibited strong expression of GFP in tumor cells (Figure 6A, top-left). Treatment with a low dose of doxorubicin enhanced apoptosis in Hph-1-gp70-injected SCID mice but not in Hph-1-GFP-injected mice (Figure 6B and 6C). These results indicate that Hph-1-gp70 treatment and DNA damage induction increased apoptosis in FM3A cells *in vivo*. Next, tumor growth was analyzed *in vivo* in SCID mice treated with Hph-1-GFP or Hph-1-gp70 and doxorubicin twice a week after the establishment of the FM3A tumor mass. When mice injected with Hph-1-gp70 and doxorubicin were compared with mice injected with Hph-1-GFP and doxorubicin, the former exhibited smaller tumor sizes and slower tumor expansion (Figure 6D). A survival analysis was also performed with each experimental group of mice. Mice treated with Hph-1-gp70 and doxorubicin exhibited a significant improvement in survival when compared with the other groups of mice (Figure 6E). These results show that breast cancer therapy with Hph-1-gp70 and a low-dose of doxorubicin was effective *in vitro* and *in vivo*.

### MCM2 is highly expressed in FM3A cells with high CD133 expression

**Figure 7:**
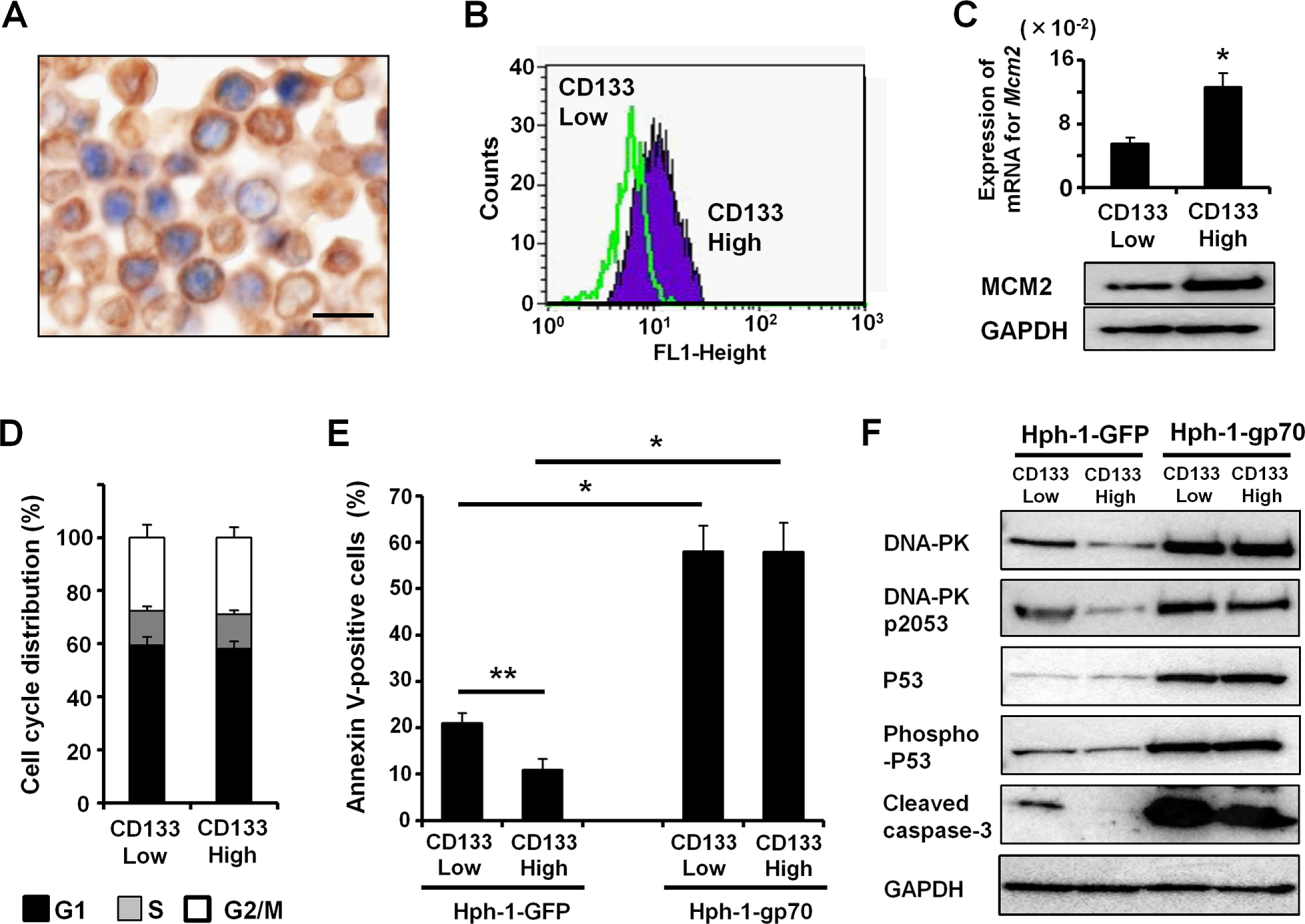
CD133-high cells have high MCM2 expression **A.** Double immunostaining for CD133 (brown) and MCM2 (blue) in FM3A cells transplanted into SCID mice. Scale bars indicate 25 μm. **B.** FACS analysis of the expression of CD133 in separated FM3A cells. **C.** Quantitative RT-PCR analysis of *Mcm2* mRNA expression (top) and western blot analysis of MCM2 (bottom) in CD133-high or -low FM3A cells. *P < 0.01 by two-tailed Student's *t*-test. **D.** The cell cycle profiles of CD133-high or -low FM3A cells were analyzed with FACS. **E.** CD133-high or -low FM3A cells were treated with 1 μM Hph-1-gp70 or Hph-1-GFP and 500 nM of doxorubicin, and the apoptotic cell ratios were determined 24 h later with annexin V-staining. *P < 0.01, **P < 0.05 by two-tailed Student's *t*-test. **F.** Representative western blots showing DNA-PK, phospho-DNA-PK (pS2053), P53, phospho-P53, and cleaved caspase-3 levels in CD133-high or -low FM3A cells treated with 1 μM Hph-1-gp70 or Hph-1-GFP and 500 nM doxorubicin for 24 h.

Next, to evaluate the relationship between the localization of CSC markers and MCM2, we performed double immunostaining for CD133 and MCM2 in FM3A transplanted SCID mice. As shown in Figure 7A, CD133 highly expressing (CD133-high) FM3A cells frequently colocalized with MCM2 *in vivo*. Therefore, to examine the expression level of MCM2 in CD133-high breast cancer cells, we separated and enriched CD133-high cells from other FM3A cells. The robust expression of CD133 in the separated FM3A cells was confirmed by FACS (Figure 7B). Approximately 7% of FM3A cells were CD133-high cells (Figure S2). As expected, the mRNA and protein levels of MCM2 were significantly higher in CD133-high FM3A cells than in CD133-low FM3A cells (Figure 7C). Moreover, CD133-high or CD133-low FM3A cells exhibited no remarkable change in their cell-cycle profiles (Figure 7D). It is widely accepted that cells expressing CSC markers have drug resistance. The present study revealed that the apoptotic cell ratio after doxorubicin treatment was significantly lower in CD133-high FM3A cells than in CD133-low FM3A cells (Figure 7E). After Hph-1-gp70 treatment, doxorubicin-induced apoptosis was enhanced in CD133-low FM3A cells. Strikingly, Hph-1-gp70-induced apoptosis was at similar levels in CD133-high and low FM3A cells (Figure 7E). Furthermore, protein levels of DNA-PK, phospho-DNA-PK (pS2053), P53, phospho-P53, and cleaved caspase-3 were lower in CD133-high FM3A cells than in CD133-low FM3A cells after treatment with doxorubicin. After Hph-1-gp70 treatment, the levels of these proteins were remarkably elevated in CD133-low and CD133-high FM3A cells (Figure 7F). These results suggest that the treatment of cells with Hph-1-gp70 and a low dose of doxorubicin strongly activate apoptosis, even in CD133-high cancer cells that were originally resistant to doxorubicin-induced apoptosis.

## DISCUSSION

The MCM complex associates with origins of DNA replication to form part of the pre-replicative complex [37]. MCM proteins are usually expressed in cycling cells [38] and are frequently overexpressed in a variety of cancer and pre-cancerous cells [23–26, 39, 40]. In breast cancer, high expression of MCM2 is associated with several clinicopathological parameters, such as advanced tumor grade, advanced stage, and poor prognosis [41, 42]. Moreover, according to an NCBI GEO database (accession number: GDS2250), the expression of *MCM2* is significantly higher in basal-like breast cancer than in normal breast cells and non-basal-like breast cancer cells. According to another NCBI GEO database (accession number: GDS4051), the expression of *MCM2* is higher in the tamoxifen-resistant breast cancer cell line MCF7 than in tamoxifen-sensitive MCF7 cells. Among the four molecular subtypes that we analyzed, the expression of MCM2 was particularly high in TNBC. These results suggest that cancer therapy targeting MCM2 will be useful for TNBC, the subtype that has lacked a specific therapeutic target.

Previous studies have suggested that the phase of the cell cycle changes after MCM2 knockdown using siRNA [43–45]. In the present study, we found that gp70 bound to with MCM2 and inhibited its nuclear translocation. However, the cell cycle profiles of the murine tumor lines were not changed by gp70 transduction (Figure 4D). In the present study, Hph-1-gp70-treated MTT060562 and 3T3 cells had MCM2 cytoplasmic fractions, yet a significant amount of MCM2 still localized to the nucleus (Figure 3D, 3E). We speculate that the cell cycle did not change because of the level of MCM2 that remained in the nucleus. However, gp70 was able to retain a significant amount of MCM2 in the cytoplasm to enhance apoptosis.

p53 is mutated or inactivated with high frequency in human breast cancer tumor cells [46]. Our study used murine breast cancer cell lines FM3A and MTT060562 that have wild-type p53 (Figure S3A). However, in 8047 cells used in our previous study, p53 has the R172H mutation (Figure S3B). Indeed, the p53 mutation (R175H) is frequently found in human breast cancers and other types of cancer [47]. Our previous study showed that therapy using gp70 enhanced apoptosis in 8047 cells [30]. In the present study, we evaluated the apoptotic cell ratio of Hph-1-gp70-treated 8047 cells after treatment with doxorubicin to induce DNA damage to determine whether this treatment is effective in a mutant p53 background. Indeed, treatment with Hph-1-gp70 increased the apoptotic index of 8047 cells (Figure S3C). Therefore, even in cells harboring p53 mutations, gp70 therapy may have similar effects as in tumors with wild-type p53. In addition, many reports have showed that anticancer agents are not as effective against breast cancer cells that have mutant p53 [48, 49]. However, other reports have indicated that high dose therapy with alkylating agents is effective against breast cancer cells that have mutant p53 [50]. Thus, cancer therapy using gp70, which enhances DNA damage-induced apoptosis, might be effective against p53-mutated breast cancer cells in combination with low doses of anticancer agents.

Previous studies have reported that activated DNA-PK stabilized MDM2 leading to the prevention of p53 degradation [51]. Our study suggests that over-activated DNA-PK is associated with p53 stabilization and activation. This result was obtained from our data showing that total p53 and phosphorylated (active) p53 levels increased to a similar extent in cells treated with Hph-1-gp70.

Because CSCs are associated with recurrence or distant metastasis after cancer therapy, it is important to develop methods that target these cells in order to improve the outcome of conventional cancer therapy [14, 52, 53]. Several reports indicate that CD133 and ALDH-1 are useful for identifying CSC-like populations in different malignancies and that CSCs are potential targets of therapy [54–57]. Furthermore, MCM2 is highly expressed in CSCs of retinoblastoma and glioblastoma [58, 59]. In the present study, CD133 and ALDH-1 were expressed in several breast cancer samples. CD133 and ALDH-1 were expressed more frequently in the TNBC group, which expressed high levels of MCM2, than in the other cancer subtypes. Immunohistochemical analysis revealed that MCM2 colocalized with CD133 in TNBC cells. Furthermore, MCM2 expression was higher in CD133-high FM3A cells than in CD133-low FM3A cells. Thus, therapies targeting MCM2-targeting may be effective against TNBC cells and CSC-like breast cancer cells.

Cancer stem cells are considered a very small fraction of cells in the cancer cell population. In the present study, CD133 was positive in about 7% of murine breast cancer cell line FM3A (Figure S2). Previous studies demonstrated that cells expressing CD133 or ALDH-1 have a stemness property [60–62]. On the other hand, our study and previous studies showed that CD133 and ALDH-1 are frequently expressed in various cancer types [63–66]. However, CD133 or ALDH-1 positive cancer cells may not necessarily possess a stemness property. Instead, these cells may have a relatively poorly differentiated character and stem cell-like phenotype. These cells may be “immature” tumor cells having stemness functions including drug resistance.

To develop a novel method for the transduction of gp70 into cancer cells, we focused on a protein delivery system that uses the PTD. The discovery of the PTD has made it possible to transduce therapeutically active agents into living cells [67, 68]. The most extensively studied PTDs are those of the HIV-1 transactivator of transcription protein (TAT), *Drosophila* homeodomain transcription factor Antennapedia (ANTP), and herpes simplex virus structural protein VP22 [69–73]. These PTD sequences are enriched with arginine residues, and short sequences with 6–30 consecutive arginine residues are functional PTDs [74–76]. Recently, a novel PTD was identified in the human transcriptional factor Hph-1. A previous study showed that Hph-1-PTD has good protein transduction efficiency and can deliver therapeutic protein *in vitro* and *in vivo* [35, 36, 77]. In the present study, we conjugated Hph-1-PTD to gp70 (Hph-1-gp70). *In vitro*, Hph-1-gp70 was rapidly and effectively introduced into breast cancer cell lines, in which it bound to MCM2 in the cytoplasm and enhanced DNA damage-induced apoptosis in cells expressing high levels of MCM2. Moreover, in an FM3A-bearing mouse model, gp70 was introduced into tumor cells through an intraperitoneal injection of Hph-1-gp70. Mice injected with Hph-1-gp70 and treated with doxorubicin exhibited a smaller tumor size and significant improvement in survival. Moreover, similar results were obtained in experiments using the ovarian cancer cell line T-Ag-MOSE (Supplementary Figure S4 and S5). These results suggest that Hph-1-gp70 offers a novel strategy for the treatment of breast cancer and other types of cancer.

In a previous study, we showed that the cytoplasmic localization of the MCM2-gp70 complex promoted the interaction of MCM2 with PP2A [30]. In this study, Hph-1-gp70 and MCM2 complex bound to PP2A (Figure 5D). PP2A has been shown to dephosphorylate DNA-PK and inhibit its function [78–80]. These results suggested that the Hph-1-gp70 and MCM2 complex bound to PP2A and inhibited the function of PP2A. Consequently, DNA-PK was hyperphosphorylated and enhanced doxorubicin-induced apoptosis via a p53/cleaved caspase 3 pathway.

Recent progress in CSC research has revealed the molecular mechanisms of resistance to chemotherapy and radiotherapy and has contributed to the development of effective chemotherapeutic regimens [81]. Several studies have demonstrated an association between CSCs and drug resistance in breast cancer cell lines [82, 83]. CD133-high FM3A cells were resistant to doxorubicin-induced apoptosis in the present study. After Hph-1-gp70 treatment, doxorubicin-induced apoptosis was enhanced not only in CD133-low FM3A cells but also in CD133-high cells. These results suggest that Hph-1-gp70 and low-dose doxorubicin treatment is an effective approach for killing cancer cells including CSCs, the treatment of which might prevent cancer relapse and metastasis. In the clinical setting for breast cancer therapy, neoadjuvant chemotherapy (NAC) regimens usually include doxorubicin or its derivatives [83, 84]. Thus, a therapeutic strategy using Hph-1-gp70-MCM2 might be a novel and effective NAC approach to treat breast cancer.

In conclusion, our data suggest that cancer therapy using Hph-1-gp70 and doxorubicin is effective for treating cancers with high MCM2 expression, including CSCs. Although several problems must be solved with regard to its application in humans, cancer therapy that exploits the apoptosis-enhancing effect of MCM2 might offer a cure for various cancers.

## MATERIALS AND METHODS

### Clinical samples

Formalin-fixed, paraffin-embedded (FFPE) samples of invasive carcinoma of no special type (ductal-NST) [85, 86] were used in this study. The molecular subtypes of breast cancer were defined using the immunohistochemical surrogate markers ER, PgR, and HER2. The HER2 subtype was defined as ER-, PgR-, and HER2+. The luminal subtype was defined as ER+ and/or PgR+ and HER2-. The luminal/HER2 subtype was defined as ER+ and/or PgR+ and HER2+. The TN subtype was defined as ER-, PgR-, and HER2-. In total, 104 FFPE samples of invasive carcinoma of no special type were collected from breast cancer patients who underwent surgery, including 30 patients with the HER2 subtype (median age, 56.5 [range, 33–76 years]), 25 patients with the luminal subtype (median age, 51 [range, 23–81 years]), 29 patients with the luminal/HER2 subtype (median age, 48 [range, 29–73 years]), and 20 patients with the TN subtype (median age, 58 [range, 34–80 years]). The FFPE samples were collected at the Cancer Institute Hospital, Tokyo, Japan in 2012. A list of the samples used in this study is summarized in Supplementary Table S1. The human study was approved by the ethics committees of Tokyo Medical and Dental University (No. 1458) and Cancer Institute Hospital (No. 2014–1003), and all procedures were performed in accordance with the ethical standards established by these committees. The experiments conformed to the principles set out in the WMA Declaration at Helsinki and the Department of Health and Human Services Belmont Report.

### Immunohistochemistry

FFPE tissue sections (4-μm thick) were used for immunohistochemistry. Deparaffinization was followed by heat-based antigen retrieval, endogenous peroxidase blockade with 3% hydrogen peroxide, and blocking with normal sera. The primary antibodies used were as follows: MCM2, mouse monoclonal, 1:2000 (BD Biosciences, San Jose, CA, USA); CD133, mouse monoclonal, 1:100 (Miltenyi Biotec, Auburn, CA, USA); ALDH-1, rabbit monoclonal, 1:1000 (Abcam, Cambridge, UK); FLAG, mouse monoclonal, 1:250 (InvivoGen, San Diego, CA, USA); and GFP, mouse monoclonal, 1:100 (Abcam). The primary antibodies were incubated overnight at 4°C. Primary antibodies were detected using an ABC Kit (Vector Laboratories, Burlingame, CA, USA) or EnVision^+^ System-HRP (Dako, Glostrup, Denmark) with diaminobenzidine (DAB; Nichirei Bioscience, Japan) or the HISTOFINE simple stain AP series (Nichirei Bioscience) with Vector Blue (Vector Laboratories). A TUNEL assay was also performed using the *In Situ* Cell Death Detection Kit, POD (Roche Diagnostics, Tokyo, Japan) with DAB. For double immunostaining, samples were heat treated and blocked between each step.

### Cell lines

The FM3A cell line derived from C3H mouse breast cancer cells was purchased from the JCRB Cell Bank (Ibaraki, Osaka, Japan) and cultured in MEM (Wako, Tokyo, Japan). 3T3 cells derived from BALB/c mouse fibroblast cells were purchased from the RIKEN Cell Bank (Tsukuba, Ibaraki, Japan) and cultured in DMEM (Sigma Aldrich, St. Louis, MO, USA). The MTT060562 cell line derived from C3H mouse breast cancer cells and T-Ag-MOSE cell line derived from C3H mouse ovarian cancer cells were purchased from the JCRB Cell Bank (Ibaraki, Oosaka, Japan) and cultured in DMEM (Sigma Aldrich). The radiation-induced myeloid leukemia cell line from C3H mice, 8047, was established at the National Institute of Radiological Sciences in Chiba [29]. The cells were cultured in RPMI-1640 medium (Sigma Aldrich). The data of T-Ag-MOSE and 8047 cell lines were shown in Supplementary Figures. Medium was supplemented with 10% fetal calf serum, penicillin (50 units/mL; Invitrogen, Carlsbad, CA, USA), and streptomycin (50 μg/mL; Invitrogen). The cells were cultured at 37°C in a humidified atmosphere of 5% CO_2_.

### Generation of Hph-1 fusion proteins to introduce proteins into cells

Gp70 was amplified from the cDNA of FLV-infected 8047 cells by using PCR primers. The primers, synthesized at a commercial laboratory (Invitrogen), were GAAAGATCTAAAAGGTCCAGCGTTCTCAAA and GAAGGTACCCTATGCAGCTATGCCGCCCATAG. GFP was amplified from the pSUPER vector (Oligoengine, Seattle, WA, USA) by using PCR primers. The primers, synthesized at a commercial laboratory (Invitrogen), were GAAAGATCTGCCACAACCATGGTGAGCAAG and CCCAAGCTTCTACTTGTACAGCTCGTCCATGCC. The PCR products were subsequently cloned into the bacterial recombinant protein expression vector pRSET C (Invitrogen). Hph-1-gp70 and Hph-1-GFP proteins contained the sequence for 2 × Hph-1-PTD (YARVRRRGPRP YARVRRRGPRP) at their N-terminus. The FLAG sequence was inserted between the Hph-1 and gp70 sequences. gp70 lacking Hph-1-PTD was also constructed and purified for use as a negative control.

The protein expression vectors were transformed into *Escherichia coli* BL21 Star (DE3)pLysS cells (Invitrogen) and cultured in Luria-Bertani medium containing 100 μg/mL ampicillin and 35 μg/mL chloramphenicol. Protein expression was induced for 2 h at 37°C by adding 1 mM isopropyl-β-d-thiogalactopyranoside. Cells were then sonicated in lysis buffer (6 M urea, 20 mM Tris-HCl, pH 8.0, and 500 mM NaCl). The lysates were clarified by centrifugation, and cOmplete His-tag Purification Resin (Roche) was added. Bound proteins were washed and eluted with 300 mM imidazole. Eluted proteins were desalted using PD-10 Sephadex G-25 (BD Biosciences), supplemented with 10% glycerol, and frozen at −80°C for later use.

### Immunoblotting

FM3A, MTT060562, and 3T3 cells, before and after treatment with transduction proteins, were used for western blotting. The samples were loaded onto a 5%–20% gradient polyacrylamide gel (Wako, Tokyo, Japan) and electrophoretically transferred to nitrocellulose membranes (GE Healthcare, Danbury, CT, USA). The membranes were blocked with 10% skim milk in PBS. The primary antibodies were anti-FLAG M2 mouse monoclonal antibody (Sigma Aldrich), anti-HA mouse monoclonal antibody (Sigma Aldrich), anti-MCM2 mouse monoclonal antibody (BD Biosciences), anti-DNA-PK_cs_ mouse monoclonal antibody (Santa Cruz Biotechnology, Santa Cruz, CA, USA), anti-DNA-PK S2056 (Mouse-S2053) rabbit polyclonal antibody (Assay Biotech, Sunnyvale, CA, USA), anti-P53 mouse monoclonal antibody (Merck, Darmstadt, Germany), anti-phospho-P53 (Ser 15) rabbit polyclonal antibody (Merck), anti-cleaved caspase-3 rabbit monoclonal antibody (Cell Signaling Technology, Danvers, MA, USA), anti-GFP mouse monoclonal antibody (Abcam), anti-PP2A rabbit polyclonal antibody (Cell Signaling Technology) and anti-GAPDH rabbit polyclonal antibody (Santa Cruz Biotechnology). The secondary antibodies were horseradish peroxidase (HRP)-conjugated anti-mouse IgG (GE Healthcare) and HRP-conjugated anti-rabbit IgG (GE Healthcare). Protein expression was detected using the Clarity™ Western ECL Substrate (Bio-Rad, Hercules, CA, USA).

### Immunofluorescence

To monitor the control-gp70 and Hph-1-gp70 protein in living cells, these proteins were labeled with fluorescein isothiocyanate (FITC) by using Fluorescein Labeling Kit-NH2 (Dojindo, Kumamoto, Japan). FM3A, MTT060562, and 3T3 cells were incubated with FITC-conjugated control-gp70 or Hph-1-gp70. After incubation, FM3A cells were washed PBS, and fluorescence was detected by a flow cytometer (BD FACSCanto™ Cell Analyzer; BD Biosciences). MTT060562 and 3T3 cells were washed PBS, and incubated with Hoechst 33342 (life technologies, CA, USA). Images were acquired using a BZ-9000 microscope (KEYENCE, Osaka, Japan) with a 400 × objective.

To monitor MCM2 localization after treatment with gp70, MTT060562 and 3T3 cells were fixed in 1% paraformaldehyde in PBS and permeabilized with 0.1% NP-40 in PBS at room temperature. Cells were incubated with a rabbit monoclonal anti-FLAG antibody (InvivoGen) and a mouse monoclonal anti-HA antibody (InvivoGen) or monoclonal anti-MCM2 antibody (BD Biosciences) at a 1:100 dilution in PBS for 1 h at room temperature. Cells were then stained with a FITC-conjugated anti-rabbit antibody (Dako) and a tetramethylrhodamine- 5-(and-6)-isothiocyanate (TRITC)-conjugated anti-mouse antibody (Dako) at a 1:100 dilution for 20 min at room temperature. Slides were washed three times with PBS and mounted with VECTASHIELD mounting medium containing 4′,6-diamidino-2-phenylindole (DAPI; Vector Laboratories). Images were acquired using a BZ-9000 microscope (KEYENCE) with a 400 × objective.

### Chromatin loading assay

Chromatin loading of MCM2 was performed as described previously [30]. Briefly, FM3A cells were lysed by incubating in complete cytoskeleton (CSK) buffer (20 mM HEPES, 100 mM NaCl, 3 mM MgCl_2_, 300 mM sucrose, and 0.1% NP-40) for 15 min on ice. Cytoplasmic fractions were obtained as supernatants after low speed centrifugation (3,000 × *g*) at 4°C. Pellets were rinsed with complete CSK buffer for 10 min on ice and centrifuged again to obtain a chromatin-enriched fraction. Pellets were then sonicated for 5 s in CSK buffer and subjected to high-speed centrifugation (16,000 × *g*). The post-sonication supernatant was designated as the chromatin-bound fraction.

### Immunoprecipitation

Cell lysates were prepared by incubating cell pellets on ice for 1 h in ice-cold lysis buffer containing 10 mM Tris-HCl, pH 7.5, 5 mM EDTA, 1% Nonidet P-40, 0.02% NaN_3_, 1 mM PMSF, 0.1% aprotinin, 100 μM leupeptin, and 100 μM TPCK (Sigma Aldrich). Cell lysates were incubated with antibody and Protein G Sepharose™ (GE Healthcare). Whole cell lysates or immunoprecipitates obtained after centrifugation were mixed with 2 × sodium dodecyl sulfate (SDS) buffer (125 mM Tris-HCl at pH 6.8, 4% SDS, 20% glycerol, 0.01% bromophenol blue, and 10% 2-mercaptoethanol) and boiled for 10 min.

### Transfection of expression plasmids

The 3 × HA-conjugated MCM2 expression vector was constructed as described previously [30]. The *3 ×HA-Mcm2* construct was transfected into 3T3 cells (2 × 10^5^ cells) using HilyMax Transfection Reagent (Nippon Gene, Tokyo, Japan). The controls were mock transfected with an empty vector.

### Detection of apoptotic cells

To determine the apoptotic cell ratios in FM3A, MTT060562, and 3T3 cells after treatment with 500 nM doxorubicin for 24 h, samples were stained with propidium iodide (PI), incubated with FITC-labeled anti-annexin V antibody (BD Biosciences), and analyzed on a flow cytometer (BD FACSCanto™ Cell Analyzer; BD Biosciences).

### Analysis of cell cycle distribution

Cell cycle distribution was monitored by quantifying the cellular DNA content after staining with PI. Cells were fixed with ethanol for 20 min at − 20°C. After centrifugation, cells were suspended in PBS containing PI (50 μg/mL) and RNase (0.2 mg/mL), incubated at room temperature for 30 min, and analyzed on a flow cytometer (BD FACSCanto™ Cell Analyzer).

### RNA interference

The siRNA sequence for *si-Mcm2* was CAGGTGACAGACTTTATCAAA. An irrelevant siRNA (*si-Control*; GCACACAGACTGCAATCACAGGTTA) that did not lead to the specific degradation of any cellular mRNA was used as a negative control. FM3A and MTT060562 cells (2 × 10^5^ cells) were transfected with 120 pmol of *Mcm2* or control siRNA by using the Amaxa^®^ Cell Line Nucleofector^®^ Kit V (Lonza, Basel, Switzerland) according to the manufacturer's instructions.

### SYBR Green real-time RT-PCR

RNA was extracted from FM3A, MTT060562 and 3T3 cell lines by using TRIzol (Invitrogen) according to the manufacturer's instructions. Briefly, the liquid phase was incubated with chloroform for phase separation. Total RNA was extracted using one isopropanol precipitation step and one ethanol wash. The RNA pellet was diluted in RNase- and DNase-free water (Qiagen, Hilden, Germany). cDNA was generated from the RNA using TaqMan^®^ Reverse Transcription Reagents (Applied Biosystems [ABI], Foster, CA, USA), and quantitative RT-PCR was performed. For quantitative RT-PCR, specific primers were used with the LightCycler SYBR Green master mix (Roche, Basel, Switzerland). The sequences of the primers are as follows: for *Mcm2*, GAGGATGGAGAGGAACTCATTG and ATCTTCCTCGCTGCTGTCA; for estrogen receptor (*ER*), GCGCAAGTGTTACGAAGTGG and AGCACCCATTTCATTTCGGC; for progesterone receptor (*PgR*), CAGAAAGGGGTTGTCCCCAG and TTCCGGAAATTCCACAGCCA; and for *Her2*, CCTGTCGACATGGACACCAA and GGACTCTCACCCCAACAGTG. The PCR product was detected using an ABI Prism 7900HT Sequence Detection System (ABI, Carlsbad, CA, USA). The primers and TaqMan probes for *Gapdh* were purchased from ABI. *Mcm2*, *ER*, *PgR* and *Her2* RNA levels were normalized to the level of *Gapdh*.

### Separation of CD133-high cells among FM3A cells

CD133-positive cells were separated from FM3A cells using a pluriBead kit (pluriSelect, San Diego, CA, USA) according to the manufacturer's instructions. Briefly, the total population of FM3A cells was incubated with 50 μL of beads directly conjugated to rabbit anti-mouse CD133 antibody (Abnova, Taipei, Taiwan) at room temperature for 30 min. The suspended cells were added to a cell strainer. The labeled CD133-high cells were retained on the cell strainer, and the unlabeled cells were eluted. Subsequently, the separated cells were incubated with FITC-labeled anti-mouse CD133 antibody (eBioscience, San Diego, CA USA) and analyzed on a flow cytometer (BD FACSCanto™ Cell Analyzer).

### Cancer-bearing mouse model

Six-week-old male specific-pathogen-free SCID mice (C.B.17^*scid/scid*^, *H-2^d^*) were purchased from CLEA Japan Inc. (Tokyo, Japan). To evaluate the therapeutic effect of Hph-1-gp70 and low-dose doxorubicin, FM3A cells (2 × 10^6^ cells/50 μL PBS) were inoculated subcutaneously into the left flank of mice. One week after transplantation, mice were treated twice a week with an intraperitoneal injection of 5 mg of Hph-1-gp70 or Hph-1-GFP and with 1.5 mg/kg of doxorubicin. The tumor volumes were calculated weekly as (width)^2^ × length × 0.52. The animal experiments were conducted and carried out in strict accordance with the Act on Welfare and Management of Animals of the government of Japan and the Guidelines for the Care and Use of Laboratory Animals of the Tokyo Medical and Dental University. All experiments were approved by the Animal Experiment Committee of Tokyo Medical and Dental University (No. 100115). The animals were housed in standard cage at 25°C, in a 12/12 light-dark cycle in a clean room. All efforts were made to minimize suffering in animal experiments.

### Statistical analysis

Data are presented in the figures as the mean ± SD. For each figure, statistical tests are justified as appropriate. The Mann-Whitney *U*-test was used to analyze the immunohistochemistry data obtained using human invasive breast cancer samples. For Kaplan-Meier analysis of SCID mice transplanted with FM3A cells, a log-rank test was performed. The statistical significance in *in vitro* experiments was determined using a two-tailed Student's *t*-test. *P* < 0.05 was considered statistically significant.

## SUPPLEMENTARY FIGURES AND TABLE


