# Supplementary material for: Targeting MCM2 function as a novel strategy for the treatment of highly malignant breast tumors
Source: Oncotarget. 2015 Sep 30;6(33):34892–909. doi: 10.18632/oncotarget.5408 (PMC4741497; doi:10.18632/oncotarget.5408)
Supplement: Supplementary file 1 [file oncotarget-06-34892-s001.pdf]

SUPPLEMENTARY FIGURES AND TABLE

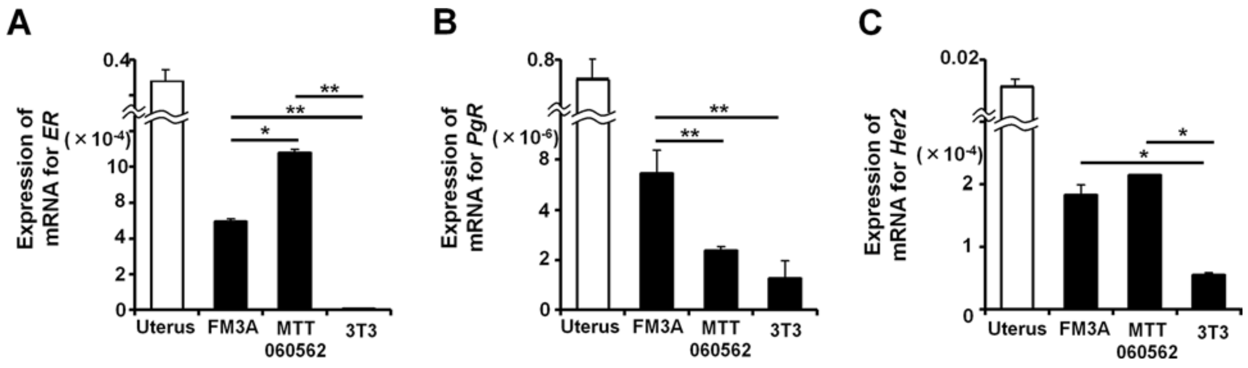

Supplementary Figure S1: Characterization of the subtype of murine breast cancer cell lines. Quantitative RT-PCR analysis of **A.** estrogen receptor (*ER*), **B.** progesterone receptor (*PgR*), **C.** *Her2* mRNA expression in FM3A, MTT060562, and 3T3 cells. Mouse uterus cDNA is shown as a positive control. The expression of *ER* mRNA was significantly higher in FM3A and MTT060562 cells than in 3T3 cells, *PgR* mRNA was significantly higher in FM3A than in 3T3 cells and *Her2* mRNA was significantly higher in FM3A and MTT060562 cells than in 3T3 cells. *$P < 0.05$, **$P < 0.01$ by two-tailed Student's *t*-test.

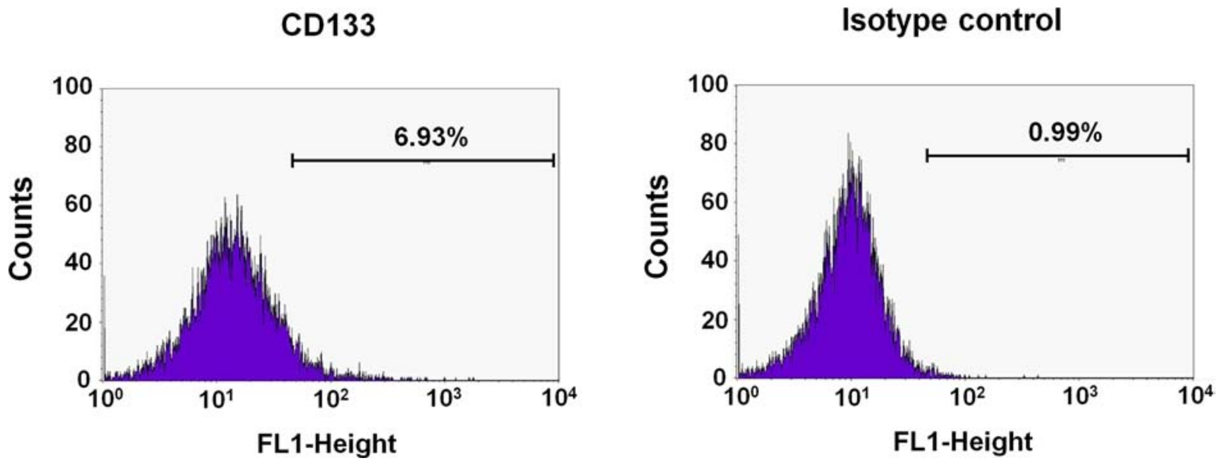

Supplementary Figure S2: CD133 expression in FM3A cells. The surface expression of CD133 measured by FACS, represent positive staining for CD133 (left) and negative control stained with matched isotype antibody (right).

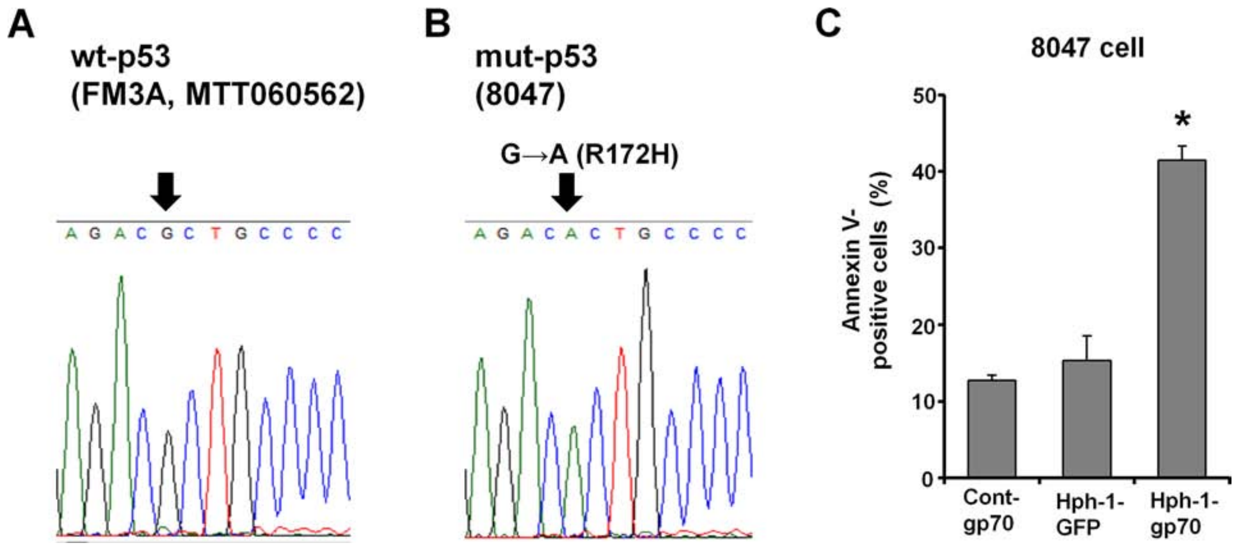

Supplementary Figure S3: Hph-1-gp70 enhances DNA damage-induced apoptosis on p53-mutated cell line. A. Sanger sequence of wild type p53 (wt-p53) was identified in FM3A and MTT060562 cells. **B.** Sanger sequence of mutated p53 (mut-p53, R172H) was identified in 8047 cells. **C.** 8047 cells were treated with 1 μM cont-gp70, Hph-1-GFP, or Hph-1-gp70 protein and 500 nM doxorubicin, and the apoptotic cell ratios were determined 24 h later with annexin V-staining. Note the significant increase in the apoptotic cell ratio after treatment with doxorubicin and Hph-1-gp70. $^*P < 0.01$ for each group by two-tailed Student's *t*-test.

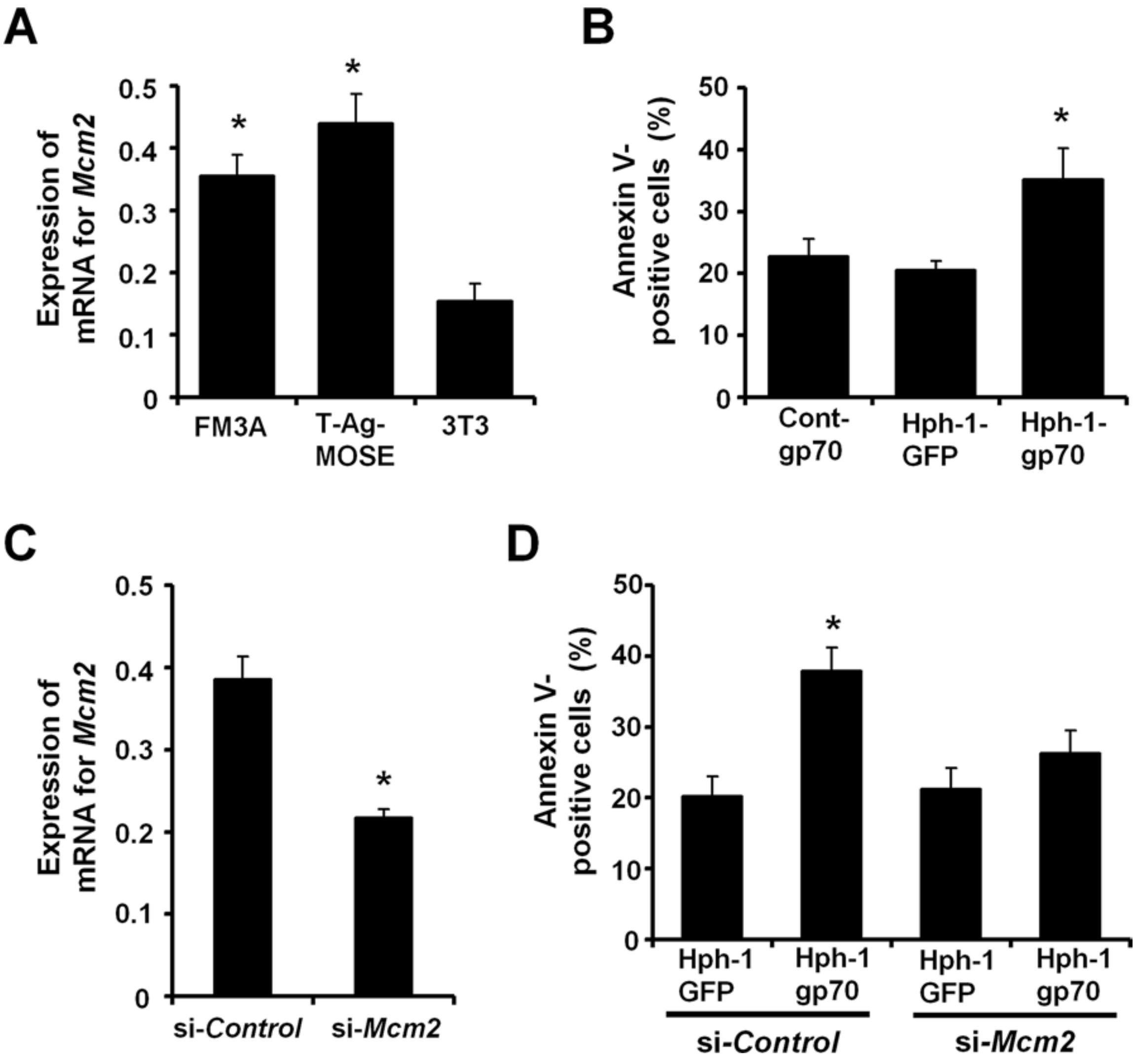

Supplementary Figure S4: Hph-1-gp70 enhances doxorubicin-induced apoptosis in T-Ag-MOSE ovarian cancer cells. A. Quantitative RT-PCR analysis of *Mcm2* mRNA expression in FM3A, T-Ag-MOSE, and 3T3 cells. **P* < 0.01 vs. 3T3 cells. **B.** T-Ag-MOSE cells were treated with 1 μM Cont-gp70, Hph-1-GFP, or Hph-1-gp70 protein and 500 nM doxorubicin, and the apoptotic cell ratios were determined 24 h later with annexin V-staining. Note the significant increase in the apoptotic cell ratio after treatment with doxorubicin and Hph-1-gp70. **P* < 0.01 for each group by two-tailed Student's *t*-test. **C.** *Mcm2* knockdown in T-Ag-MOSE cells using siRNA. Quantitative RT-PCR was performed to confirm the *si-Mcm2*-induced reduction of *Mcm2* mRNA expression. **P* < 0.01 by two-tailed Student's *t*-test. **D.** T-Ag-MOSE cells transduced with *Si-control* or *si-Mcm2* were treated with 1 μM Hph-1-GFP or Hph-1-gp70 and 500 nM of doxorubicin, and the apoptotic cell ratios were determined 24 h later with annexin V-staining. Note that the apoptotic induction triggered by doxorubicin and Hph-1-gp70 was inhibited by *si-Mcm2* treatment (**P* < 0.01 by two-tailed Student's *t*-test).

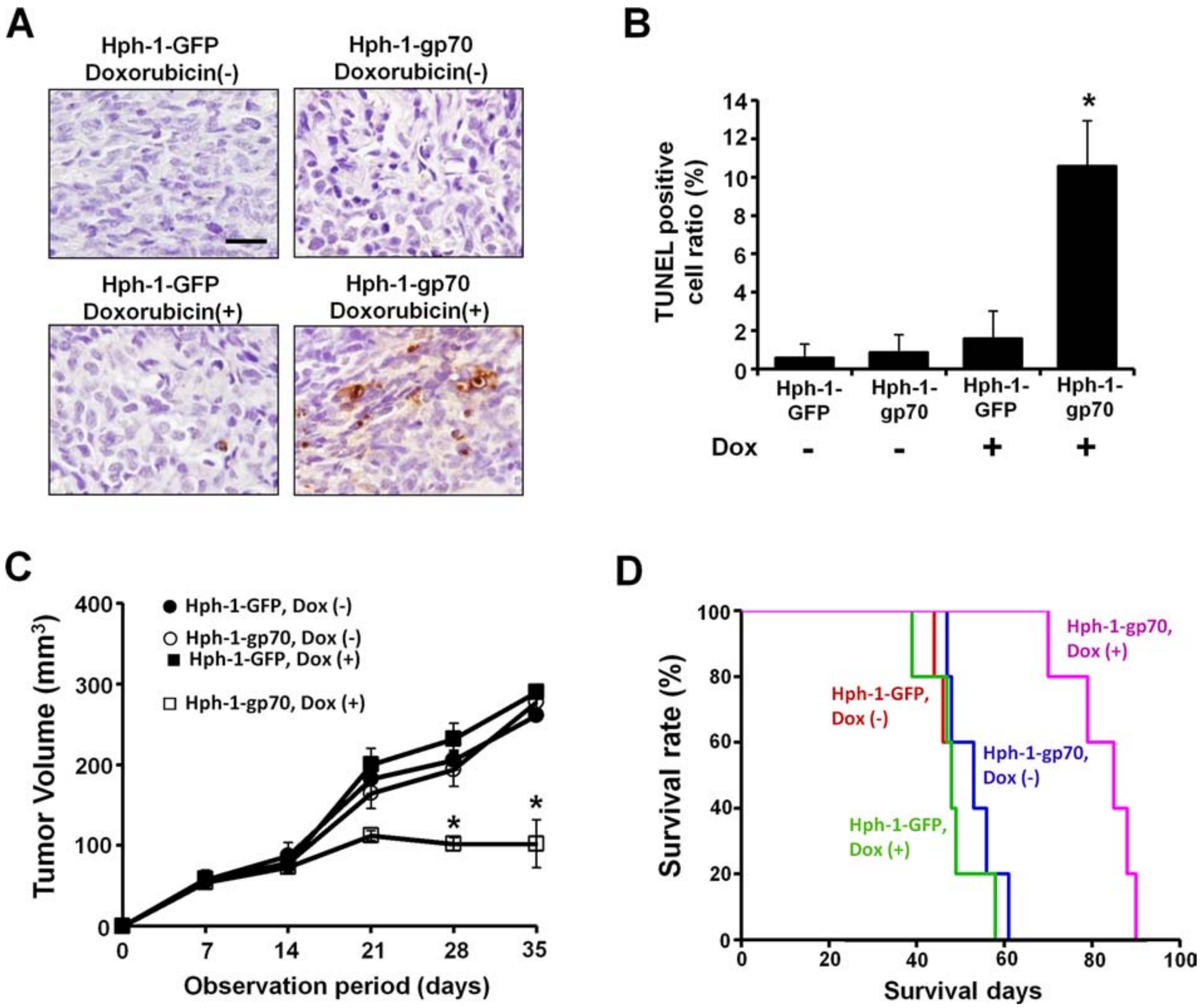

Supplementary Figure S5: ***In vivo*** **anti-tumor effects of Hph-1-gp70 and DNA-damage in T-Ag-MOSE cells in SCID mice. A.** Seven days after the transplantation of T-Ag-MOSE cells, mice were treated with Hph-1-gp70 or Hph-1-GFP and 1.5 mg/kg of doxorubicin or PBS. Microscopic features of TUNEL-positive cells in a T-Ag-MOSE tumor. **B.** TUNEL-positive cell ratio in each group of mice. Scale bars indicate 100 μm. Note the significant induction of apoptosis after treatment with doxorubicin and Hph-1-gp70 (*$P < 0.01$ by two-tailed Student's *t*-test). **C.** To evaluate the therapeutic effect of Hph-1-gp70 and low-dose doxorubicin, T-Ag-MOSE cells were transplanted into SCID mice. Seven days after the transplantation, mice were treated with 5 mg Hph-1-gp70 or Hph-1-GFP and 1.5 mg/kg of doxorubicin or PBS twice a week. The tumor size in each mouse was assessed once a week. *$P < 0.05$ by two-tailed Student's *t*-test. **D.** Kaplan-Meier survival curves for T-Ag-MOSE-transplanted SCID mice treated with Hph-1-gp70 or Hph-1-GFP and doxorubicin. The survival time of the Hph-1-gp70, doxorubicin (+) group was significantly longer than that of other groups ($P < 0.01$ by log-rank test).

Supplementary Table S1: List of samples used in the study

Subtype	Age	Tissue type	MCM2 (%)	CD133	CD133/MCM2 colocalization	ALDH-1	ALDH-1/MCM2 colocalization
HER2	64	Scirrhous carcinoma	100	−		−	
HER2	67	Solid-tubular carcinoma	100	−		−	
HER2	57	Scirrhous carcinoma	50	−		+	○
HER2	50	Papillotubular carcinoma	50	cytoplasmic	×	−	
HER2	53	Scirrhous carcinoma	100	cytoplasmic	○	+	○
HER2	55	Scirrhous carcinoma	100	cytoplasmic	×	+	○
HER2	67	Papillotubular carcinoma	70	membranous	○	+	○
HER2	64	Solid-tubular carcinoma	90	−		−	
HER2	54	Solid-tubular carcinoma	90	−		−	
HER2	76	Solid-tubular carcinoma	100	−		−	
HER2	73	Scirrhous carcinoma	50	−		−	
HER2	56	Scirrhous carcinoma	80	cytoplasmic	○	−	
HER2	65	Solid-tubular carcinoma	90	−		+	○
HER2	63	Solid-tubular carcinoma	100	−		−	
HER2	33	Papillotubular carcinoma	90	cytoplasmic	○	−	
HER2	49	Scirrhous carcinoma	100	cytoplasmic	○	−	
HER2	56	Solid-tubular carcinoma	70	−		−	
HER2	55	Solid-tubular carcinoma	100	−		−	
HER2	59	Solid-tubular carcinoma	90	−		+	○
HER2	48	Solid-tubular carcinoma	80	−		−	
HER2	76	Scirrhous carcinoma	100	−		−	
HER2	60	Solid-tubular carcinoma	90	−		−	
HER2	51	Scirrhous carcinoma	30	membranous	×	−	
HER2	52	Scirrhous carcinoma	100	cytoplasmic	○	−	○
HER2	53	Scirrhous carcinoma	100	−		−	
HER2	58	Solid-tubular carcinoma	90	−		−	
HER2	62	Solid-tubular carcinoma	50	−		−	
HER2	50	Papillotubular carcinoma	90	−		−	
HER2	53	Solid-tubular carcinoma	80	−		−	
HER2	76	Solid-tubular carcinoma	100	−		+	○
Luminal	62	Solid-tubular carcinoma	50	cytoplasmic	×	−	
Luminal	46	Papillotubular carcinoma	10	−		−	

(*Continued*)

Subtype	Age	Tissue type	MCM2 (%)	CD133	CD133/MCM2 colocalization	ALDH-1	ALDH-1/MCM2 colocalization
Luminal	50	Papillotubular carcinoma	50	−		−	
Luminal	61	Scirrhous carcinoma	10	−		−	
Luminal	53	Papillotubular carcinoma	80	−		−	
Luminal	56	Scirrhous carcinoma	10	−		−	
Luminal	56	Solid-tubular carcinoma	70	−		+	×
Luminal	52	Solid-tubular carcinoma	10	−		−	
Luminal	30	Solid-tubular carcinoma	90	−		−	
Luminal	77	Papillotubular carcinoma	10	−		−	
Luminal	51	Solid-tubular carcinoma	70	−		−	
Luminal	38	Papillotubular carcinoma	10	−		−	
Luminal	31	Scirrhous carcinoma	100	−		−	
Luminal	48	Solid-tubular carcinoma	20	−		−	
Luminal	50	Papillotubular carcinoma	10	−		−	
Luminal	61	Scirrhous carcinoma	50	−		−	
Luminal	49	Papillotubular carcinoma	10	−		−	
Luminal	64	Scirrhous carcinoma	50	cytoplasmic	×	+	×
Luminal	47	Solid-tubular carcinoma	50	−		−	
Luminal	39	Papillotubular carcinoma	30	−		+	×
Luminal	23	Papillotubular carcinoma	50	−		−	
Luminal	50	Papillotubular carcinoma	10	−		−	
Luminal	51	Scirrhous carcinoma	10	−		−	
Luminal	81	Papillotubular carcinoma	50	−		−	
Luminal	70	Scirrhous carcinoma	80	−		−	
Luminal/HER2	44	Papillotubular carcinoma	70	−		−	
Luminal/HER2	29	Solid-tubular carcinoma	90	−		+	○
Luminal/HER2	47	Solid-tubular carcinoma	70	cytoplasmic	○	−	
Luminal/HER2	54	Papillotubular carcinoma	70	−		−	
Luminal/HER2	43	Scirrhous carcinoma	95	−		−	

(*Continued*)

Subtype	Age	Tissue type	MCM2 (%)	CD133	CD133/MCM2 colocalization	ALDH-1	ALDH-1/MCM2 colocalization
Luminal/HER2	51	Scirrhous carcinoma	90	−		+	○
Luminal/HER2	73	Scirrhous carcinoma	50	−		−	
Luminal/HER2	48	Papillotubular carcinoma	100	−		−	
Luminal/HER2	56	Solid-tubular carcinoma	70	−		+	○
Luminal/HER2	59	Scirrhous carcinoma	70	−		−	
Luminal/HER2	55	Scirrhous carcinoma	90	cytoplasmic	○	+	○
Luminal/HER2	40	Scirrhous carcinoma	100	−		−	
Luminal/HER2	43	Papillotubular carcinoma	70	cytoplasmic	×	−	
Luminal/HER2	40	Scirrhous carcinoma	50	−		−	
Luminal/HER2	61	Papillotubular carcinoma	80	cytoplasmic/ membranous	×	−	
Luminal/HER2	43	Scirrhous carcinoma	90	cytoplasmic	○	−	
Luminal/HER2	40	Scirrhous carcinoma	80	−		−	
Luminal/HER2	34	Scirrhous carcinoma	50	−		−	
Luminal/HER2	64	Scirrhous carcinoma	90	−		−	
Luminal/HER2	57	Solid-tubular carcinoma	70	cytoplasmic	○	−	
Luminal/HER2	46	Scirrhous carcinoma	90	cytoplasmic	×	−	
Luminal/HER2	38	Solid-tubular carcinoma	70	−		−	
Luminal/HER2	53	Scirrhous carcinoma	50	−		−	
Luminal/HER2	51	Scirrhous carcinoma	100	−		−	
Luminal/HER2	55	Scirrhous carcinoma	70	cytoplasmic	×	−	
Luminal/HER2	35	Solid-tubular carcinoma	100	−		−	
Luminal/HER2	33	Papillotubular carcinoma	30	−		+	○
Luminal/HER2	66	Solid-tubular carcinoma	80	−		−	
Luminal/HER2	53	Scirrhous carcinoma	80	−		−	
TN	80	Solid-tubular carcinoma	100	−		−	
TN	34	Solid-tubular carcinoma	80	−		−	
TN	80	Solid-tubular carcinoma	90	cytoplasmic	○	−	
TN	66	Papillotubular carcinoma	100	cytoplasmic/ membranous	○	−	
TN	56	Solid-tubular carcinoma	100	cytoplasmic	○	+	○
TN	48	Solid-tubular carcinoma	100	−		−	
TN	70	Papillotubular carcinoma	90	membranous	○	−	
TN	45	Solid-tubular carcinoma	90	−		−	

(*Continued*)

Subtype	Age	Tissue type	MCM2 (%)	CD133	CD133/MCM2 colocalization	ALDH-1	ALDH-1/MCM2 colocalization
TN	34	Solid-tubular carcinoma	100	−		−	
TN	43	Solid-tubular carcinoma	70	−		−	
TN	35	Solid-tubular carcinoma	100	−		+	○
TN	68	Solid-tubular carcinoma	80	cytoplasmic/ membranous ±	×	−	
TN	49	Scirrhous carcinoma	70	−		−	
TN	69	Solid-tubular carcinoma	100	cytoplasmic/ membranous ±	×	+	×
TN	63	Solid-tubular carcinoma	90	cytoplasmic	×	−	
TN	36	Solid-tubular carcinoma	70	−		+	×
TN	73	Solid-tubular carcinoma	100	−		−	
TN	53	Solid-tubular carcinoma	100	cytoplasmic	○	−	
TN	60	Scirrhous carcinoma	100	cytoplasmic	○	−	
TN	76	Scirrhous carcinoma	100	cytoplasmic/ membranous	○	+	○
